# Genetic Algorithm Optimization of Rainfall Impact Force Piezoelectric Sensing Device, Analytical and Finite Element Investigation

**DOI:** 10.3390/ma16030911

**Published:** 2023-01-18

**Authors:** Muath A. Bani-Hani, Dima A. Husein Malkawi, Khaldoon A. Bani-Hani, Sallam A. Kouritem

**Affiliations:** 1 Department of Aeronautical Engineering, Jordan University of Science and Technology, Irbid 22110, Jordan; 2 Department of Civil and Environmental Engineering, German Jordanian University, Amman 11180, Jordan; 3 Department of Civil Engineering, Fahad Bin Sultan University, Tabuk 71454, Saudi Arabia; 4 Department of Mechanical Engineering, Faculty of Engineering, Alexandria University, Alexandria 5424041, Egypt

**Keywords:** sensors, vibration, piezoelectric, micro-electrical, mechanical systems (MEMS)

## Abstract

In this paper, rainfall droplet impact force is transformed into a measurable voltage signal output via the piezoelectric material direct effect utilized for sensing purposes. The motivating sensor is utilized to measure the peak impact forces of rainfall droplets for further analysis and processing. Constructing a sense for the impact force of rainfall droplets has great implications in many real-life applications that can provide vital information regarding the amplifications of the impact force of rainfall on soil erosion, and the impact on small creatures and plants, etc. The rainfall droplet is set to collide on a very thin aluminum plate with negligible mass that can be presented geometrically as an extended segment of the proposed sensing device. The proposed sensing device is composed of a bimorph simply supported composite-piezoelectric beam that buckles due to the effect of the rain droplets’ vertical impact force. The proposed device is designed for optimal performance in terms of the amount of voltage that can be measured. This is accomplished by having the first critical buckling load of the device as less than the impact force of the rainfall droplet. Accordingly, the well-known genetic algorithm (GA) automated optimization technique is utilized in this paper to enhance the measured voltage signal. A proof mass is added to the middle of the beam to amplify the magnitude of the measured voltage signal. The voltage signal is intended to be transferred to the PC via a data acquisition system. The rainfall droplets’ peak impact forces are obtained analytically due to the nonlinear behavior of the beam using the Euler–Bernoulli thin beams assumptions. The FE model using COMSOL 6.0 Multiphysics commercial software is used to verify the analytical results.

## 1. Introduction

Recently, energy harvesting has been heavily investigated due to its role in low-power applications [1,2,3,4,5,6], structural health monitoring [7], and wireless self-powered sensors [8]. It also can be used to suppress ambient vibration [9]. Piezoelectric material is the most used mechanism for energy harvesting and sensing purposes due to its high energy density when compared with other harvesting approaches, such as electromagnetic and electrostatic approaches [4]. The piezoelectric sensing/energy harvesting mechanism is utilized by implementing the direct piezoelectric effect to generate a measurable voltage signal. This can be accomplished by converting the mechanical strain into an electric potential difference [5]. To enhance the voltage signal, it was found that piezoelectric coefficients in the *x* and *z* directions had a major effect on the voltage output when piezoelectric composite structures were used as bimorph or uni-morph [10].

Rainfall droplets’ impact forces have been under significant investigation due to their great implication in many real-life applications [11,12]. This includes soil erosion [13] and the impact on small creatures [14]. The collision mechanisms of a rainfall droplet include splashing, bouncing, or spreading [12,15,16]. Furthermore, although the rainfall is intermittent, it can be used as a power source for low-power applications, such as microelectronics and sensors. Electrical charges can be generated when a single drop hits a piezoelectric plate [17,18]. In addition, the rainfall droplet’s kinetic energy can be harvested using hydro turbines in regions that are characterized by sufficient rainfall [19,20,21].

Many analytical models were developed to predict the impact force or pressure of the rainfall droplet on different types of surfaces [22,23]. Cook et al. [11] introduced a water-hammer model to predict the impact pressure of a rainfall droplet colliding on a solid surface. Heymann et al. modified Cook’s water-hammer model and developed a two-dimensional model that showed that the impact pressure/force was three times bigger than Cook’s water-hammer model [24]. Safavi et al. have developed an analytical expression based on the energy balance method to predict the velocity and impact force of the liquid droplet. In addition, the analytical expression results were validated experimentally [25]. Bierbrauer et al. developed analytical modeling for water droplets’ impact forces on hot-galvanized steel surfaces to determine corrosion resistance [26].

Several numerical simulations have also been developed to investigate the liquid–solid interaction and its corresponding impact force [27,28,29]. For example, Adler et al. used finite element analysis to develop a 3D model and to predict the pressure and stress in water droplets when colliding on solid plates. Furthermore, Keegen et al. [30] used “Explicit Dynamics software” to develop a model of a rainfall droplet to predict the impact force when colliding on an epoxy resin plate at very high velocities of up to 140 m/s. In their most recent work, Li et al. and Zhou et al. [31], suggested a model that can predict the impact force due to the collision of a liquid droplet on a solid and elastic surface by implementing the wave and Lame equations for the droplets and the elastic solid surface, respectively. Their results were in good agreement with Heymann’s analytical model prediction. Bussmann et al. has established a 3D model to predict liquid droplet impact force onto asymmetric surfaces based on “RIPPLE” software and the simulation results were compared with photographic data [32]. Mitchill et al. experimentally studied the impact force of low-velocity 2.9 mm diameter water droplets using a piezoelectric force sensor. They also conducted numerical simulations to describe the dynamics of the solid–fluid interaction for liquid droplets impacting at high velocities [33]. Rui Li et al. conducted a numerical study of liquid droplets’ impact forces on a rigid wall [34]. Chensen Lin et al. investigated the dynamics of liquid droplet impact forces on a ring-shaped solid surface using a simulation method known as many-body dissipative particle dynamics (MDPD) [35].

Furthermore, experimental work has been conducted to further investigate the impact force due to the interaction between liquid and solids and to also validate the numerical results when compared with the analytical model predictions. For example, Portemont et al. used a transducer with a cavity to experimentally measure low-speed liquid droplets’ forces and pressures [36]. In addition, Nearing et al. [13] used piezoelectric pressure transducers to experimentally measure the impact force of water droplets. Piezoelectric sensors and transducers are proven to be the best option for experimental transient measurements due to their high-frequency response and high sensitivity. Grinspan and Gnanamoorthy predicted liquid droplets’ impact force by developing a (PVDF) piezoelectric film [37]. Jingyin Li et al. used an extremely sensitive piezoelectric force transducer to predict the impact forces of water droplets with different velocities and diameters [30]. Qin Yanzhou et al. numerically investigated the impact force of water on a channel surface by implementing the method of the volume of fluid (VOF) while varying the impact angle, surface contact angle, impact velocity, droplet’s size, and temperature [38]. Zhang Bin et al. experimentally studied the impact force of low-velocity rainfall droplets when colliding on a solid surface using a very sensitive piezoelectric force sensor recorded by a high-speed camera [39]. Basahi J. M, et al. used a piezoelectric material that generates a voltage signal due to the impact force of water droplets colliding on a piezoelectric film. The voltage signal was transmitted to a PC through a data acquisition board where it was measured and analyzed [40]. Jiang et al. used photoacoustic (PA) and piezo-ultrasound (PU) technology by employing traveling ultrasound waves to transmit energy wirelessly. The same strategy can be used to wirelessly transmit the measured voltage signal due to the impact force of water droplets colliding on a piezoelectric film [41,42,43]. According to the discussion above, it is evident that the sparse data obtained from the experimental impact force research work are very difficult to use as reference data for further analytical and numerical investigation.

The rain droplet impact force depends on the droplet’s size (diameter) and its impact velocity. Rainfall has three main types: light, moderate, and heavy. Wong et al. investigated several methods to determine rain droplet diameter using a photographic method [44]. Perera and Gunn calculated the terminal velocity of the rain droplet when the upward and downward forces acting on the water droplet were at equilibrium [45,46]. The impact velocities were determined based on data previously published in (Laws, Measurements of the fall velocities of water drops and raindrops, 1941). Mangili et al. [47] investigated the time duration from impact until the force vanishes completely and it was found to be almost 2 ms. The impact force for different types of rain droplet sizes and velocities can be computed by a model based on an inertia scenario that was proposed by Imeson et al. [48] and postulated by Soto [49].

In this paper, a proposed sensing device that is composed of a composite piezoelectric simply supported beam is presented to predict the peak impact force of rainfall droplets of the three types of rain. The impact force acting on the proposed and highly sensitive sensing device generates a proportional charge at the voltage signal output. This is due to piezoelectric materials’ sensing ability to transform small mechanical strains into a voltage signal over a wide force spectrum. Since the impact forces are very small, we introduced a nonlinear configuration of an elastic piezoelectric composite beam capable of sensing such small quantities of forces. The impact force of the rainfall droplet is to collide on a very thin aluminum plate with negligible mass that is longitudinally attached to the beam of the proposed sensing device. The proposed sensing device is expected to be axially excited and will buckle due to the effect of the axial impact force of the intermittent rainfall droplets. The transitions between the unbuckled and buckled positions result in free vibrations of the proposed device at its first natural frequency as will be illustrated later in this research. A proof mass ($M_{t}$) is added to the middle of each beam to amplify the magnitude of the calculated voltage signal. The calculated voltage signal is to be further analyzed to obtain the corresponding impact forces through a mathematical model that is developed in this work. By applying the Euler–Bernoulli beam theory to the proposed device, the nonlinear analytical model is derived in this research in addition to the electro-mechanical equations. Furthermore, the proposed device is designed for optimal performance in terms of the amount of voltage signal that can be generated. This can be achieved by making sure that the measured impact force of the rainfall droplet is higher than the first critical buckling load. To do so, the genetic algorithm (GA) automated optimization method is utilized in this paper to achieve optimal performance in terms of the generated voltage signal of the proposed device [50]. The principle of the GA optimization method is to generate random values for the design parameters to approach the global optimal [51]. The genetic algorithm (GA) method is an automated design methodology used to minimize any possible human intervention and reduce the efforts to obtain adequate results compared with other conventional optimization techniques. The well-known COMSOL 6.0 Multiphysics is used to verify the analytical results of the proposed sensing device by employing a finite element model (FEM). The COMSOL is a very powerful finite element software to solve very complicated models when it is difficult to conduct physical experiments. Due to its accuracy and reliability, it is widely used among researchers, which leads us to be very confident in our FEM results to verify the analytical model results.

## 2. Design Configuration and Mathematical Model

**Figure 1:**
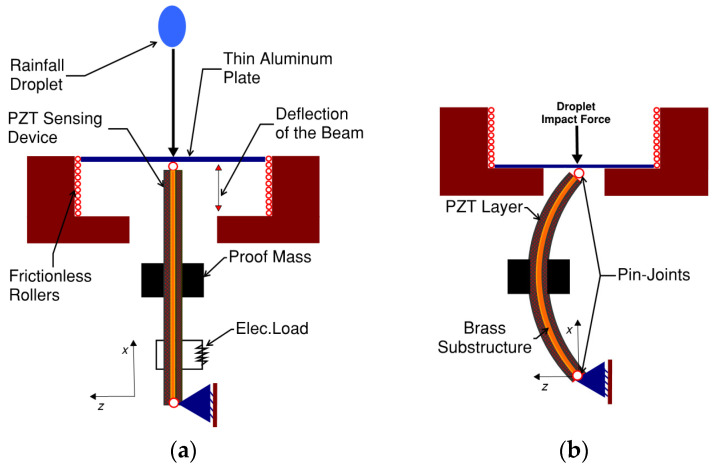
Proposed rainfall impact force sensing device (**a**) before the impact (**b**) after the impact.

The frequency of a falling rainfall droplet can vary from fractions of seconds as in downpour rainfall and can be intervals of seconds as in light rainfall [52,53]. Although the impact of rainfall droplets can take different shapes such as splashing, bouncing, or spreading [12], we are only concerned with the vertical component of the peak impact force exerted by rainfall droplets in terms of magnitude and frequency [54]. The rain droplet is to collide on a very thin aluminum plate. Figure 1a,b show the proposed device before and after the rain droplet impact on the aluminum plate, respectively.

Rainfall has 3 main types: light, moderate, and heavy, as listed in the 1st column of Table 1. Researchers have investigated different approaches to estimate rain droplet size using a photographic method [44]. Before the impact, the rain droplets are exposed to a vertical force of 2 types; drag force acting upward, and gravitational force acting downward. The terminal velocity can be calculated when the upward and downward forces are at equilibrium [45,46] as illustrated in Equation (1). The size of a single drop of water can range from 2 to 5 mm as listed in the 2nd column of Table 1 [44]. Consequently, the speed of the impact can be evaluated accordingly and can range from 6 m/s to 9 m/s as listed in the 3rd column of Table 1.

(1) $$\begin{array}{c} F_{d}=\frac{1}{2}\rho_{a}ACv_{t}^{2},\;\;\;\;\;\;F_{g}=\frac{4}{3}\pi r^{3}\rho_{w}g\\ v_{t}=\sqrt{\frac{\pi d^{3}\rho_{w}g}{6\rho_{a}AC}},\;\;\;when\;\;F_{d}=F_{g} \end{array}$$

where the air density is denoted by $\rho_{a}$, and the drag coefficient is denoted by C, $v_{t}$ is the droplet’s velocity, A is the projection of the droplet’s frontal area, $\rho_{w}$ is the rainfall droplet’s density, d is the droplet’s diameter, r is the droplet’s radius, and g is the gravitational acceleration. The impact forces of different rainfall droplets’ diameters and velocities were experimentally investigated by Soto et al. in their work [49]. It was found that the peak impact force depended on the impact velocity as per a model that was proposed based on an inertia scenario. Moreover, the measurements presented matched the numerically simulated results when the theory of potential flow was assumed as was presented in Mangili et al. [47]. Furthermore, according to their studies, the time duration of the droplet’s impact force to rise and disappear was almost 2 ms. At this point, Table 1 can now be updated to show the impact forces as listed in the 5th column.

A descriptive sketch of the sensing element of the proposed device is illustrated in Figure 1. Two positions are illustrated; (a) un-deformed shape (before the impact) and (b) deformed shape (after the impact). The piezoelectric layer generates a voltage signal when it buckles and then returns to the un-deformed shape. To realize the simply supported boundary conditions, the beam is supported by pin joints at both ends. The pin joints themselves can be realized by using bearings on either side of the proposed sensing device to employ the simply supported boundary conditions. Hence, the joints are free to rotate via the bearings. The theoretical assumptions of the buckling phenomenon can be realized by mounting one of the bearing housings on a slider or frictionless rollers to allow the sliding of one beam end in the longitudinal direction. Frictionless rollers are proposed as indicated in Figure 1a. Therefore, the device can be analytically modelled as a damped pin-pin beam using the assumptions of the Euler–Bernoulli beam theory.

More voltage output means more sensing capability. Therefore, the idea here is to design the device such that the droplet’s impact force is higher than the 1st critical load and less than the ones of higher modes of the proposed device. This is because high power cancelation occurs at higher modes. Furthermore, a proof mass ($M_{t}$) is added to the middle of the beam to amplify the magnitude of the measured voltage signal. The governing equation of the proposed device can be written in the form illustrated in (2) [55,56,57,58].

(2) $$\begin{array}{c} m\;\frac{\partial^{2}w\left(x,\;t\right)}{\partial \;t^{2}}+c\;\frac{\partial w\left(x,\;t\right)}{\partial t}+\frac{\partial^{2}M\left(x,\;t\right)}{\partial x^{2}}=0\\ m=b\left(\rho_{s}h_{s}+2\rho_{p}h_{p}\right) \end{array}$$

m is the composite structure mass per unit length, L. w (x, t)  is deflection along the *z*-axis, M (x, t) is the internal bending moment. Superscripts p and s stand for piezoelectric and substructure layers, respectively. b is the width of the proposed harvester, $h_{s}$ and $h_{p}$ are the thicknesses of the substructure and piezoelectric layers of the proposed harvester, respectively. The substructure and piezoelectric layer densities are noted by $\rho_{s}$ and $\rho_{p}$, respectively. c is the equivalent damping term of the composite cross-section due to structural viscoelasticity and viscous air damping [59,60,61,62].

The governing Equation (2) can be updated to include the nonlinear buckling effect. Therefore, by implementing Floquet theory, the dynamic response of the proposed sensing device due to the rainfall impact force can be presented as indicated in (3) [55].

(3) $$\begin{array}{c} m\;\frac{\partial^{2}w\left(x,\;t\right)}{\partial \;t^{2}}+c\;\frac{\partial w\left(x,\;t\right)}{\partial t}+\frac{\partial^{2}M\left(x,\;t\right)}{\partial x^{2}}\\ +[F_{impact}-\frac{{\left(YA\right)}_{eq}}{2L}\;\int_{0}^{L}\;{\left(\frac{\partial w\left(x,\;t\right)}{\partial x}\right)}^{2}dx\;]\;\frac{\partial^{2}w\left(x,\;t\right)}{\partial x^{2}}=0\\ {\left(YA\right)}_{eq}=2Y_{p}A_{p}+Y_{s}A_{s},\;\;\;\;\;\;\;\;\;\;\;\;\;\;A_{p}=b\;h_{p}\;\&\;A_{s}=b\;h_{s} \end{array}$$

$F_{impact}$ is the axial statics and compressive load due to the rainfall droplet’s impact force that is illustrated in Table 1. The cross-section areas of the structure are $A_{p}=b\;h_{p}$ and $A_{s}=b\;h_{s}$. The total equivalent axial stiffness of the structure is denoted by ${\left(YA\right)}_{eq}$.

In this work, the piezoelectric layers are assumed to be connected in a series, as indicated. Therefore, the partial differential governing the piezoelectric beam indicated in (4) is derived by combining the buckled beam and piezoelectric energy harvesting bimorph while implementing the standard piezoelectric constitutive relations [63] that relates the strain and stress to the electric field.

(4) $$\begin{array}{c} T_{1}^{s}=Y_{s}S_{1}^{s},\;\;\;\;\;\;\;\;\;\;T_{1}^{p}=Y_{p}\left(S_{1}^{p}-d_{31}E_{3}\right)\\ E_{3}=\frac{-V_{p}\left(t\right)}{h_{p}},\;\;V_{p}\left(t\right)=\frac{V\left(t\right)}{2}\; \end{array}$$

T and S denote the mechanical stress and strain, respectively. Y is the modulus of elasticity; $d_{31}$ is the piezoelectric strain constant that relates the electric field, $E_{3}$ produced by the mechanical stress [23]. The subscripts ‘1’ and ‘3’ represents the coordinates axis ‘*x’* and ‘*z*’, respectively. The voltage across each piezoelectric layer is denoted by $\;V_{p}\left(t\right)$. The voltage across the electric resistive load $\;R_{l}$ is denoted by V(t) illustrated in Figure 1. The piezoelectric layers are connected in series, and both layers have opposite $d_{31}$ signs so that no cancellation in the electric field $E_{3}$ can occur. This is accomplished by having the top and bottom piezoelectric layers in the same direction as indicated in (4). The internal bending moment is derived by integrating the 1st moment of the stress over the cross-sectional area as derived in [64]. The internal moment expression is then used in the partial differential equation of motion given by (2) to reach its final form as indicated in (5).

(5) $$\begin{array}{c} m\;\frac{\partial^{2}w}{\partial \;t^{2}}+c\;\frac{\partial w}{\partial t}+YI\;\frac{\partial^{4}w}{\partial x^{4}}+[F_{impact}-\frac{{\left(YA\right)}_{eq}}{2L}\;\int_{0}^{L}\;{\left(\frac{\partial w}{\partial x}\right)}^{2}dx\;]\;\frac{\partial^{2}w}{\partial x^{2}}\\ +\vartheta \left[\;\frac{d\delta \left(x\right)}{dx}-\frac{d\delta \left(x-L\right)}{dx}\right]V\left(t\right)=0\;\;\; \end{array}$$

where YI is the total and equivalent bending stiffness of the composite structure and ϑ is the electrical–mechanical coupling coefficient. Their formulas can be expressed as derived in [64]. δ(x) is the Dirac delta function. Implementing the standard piezoelectric constitutive relations [27], the voltage V(t) through the electrical load $\;R_{l}$ can be carved out as indicated in (6).

(6) $$C_{p}\;\dot{V}\left(t\right)+\frac{V\left(t\right)}{R_{l}}=\vartheta \frac{d}{dt}\left[\overset{L}{\underset{0}{\int }}\frac{\partial^{2}w\left(x,t\right)}{\partial x^{2}}\;\partial x\;\right],\;\;\;\;\;\;\;\;\;\;C_{p}=\frac{\varepsilon_{33}^{s}bL}{2h_{p}}$$

$C_{p}$ is defined in the literature by the capacitance of the PZT layers and $\varepsilon_{33}^{s}$ is the PZT permittivity constant. Equation (7) indicates an infinite series of Eigen functions that are used as a standard solution for the partial differential Equation (5).

(7) $$w\left(x,t\right)=\overset{\infty }{\underset{j=1}{\sum }}\phi_{j}\left(x\right)\;T_{j}\left(t\right)$$

The partial differential equation can be solved by using the so-called method of separation of variables. $\phi_{j}$ is jth natural mode shape and $T_{j}$ is the temporal function. It must be noted that only the first buckling mode is considered in our analysis (i.e., j=1). That is, the droplet’s impact force  F should be larger than the 1st critical load $F_{c}$ as estimated in (8).

(8) $$F_{c}=\frac{\int_{0}^{L}\phi_{j}{\;}^{\prime \prime }\left(x\right)\;\phi_{j}\left(x\right)dx}{\int_{0}^{L}\;\phi_{j}\left(x\right)\;YI\;\phi_{j}\prime \prime \prime \prime \left(x\right)dx},\;\;j=1,\;\;\;\;F_{c}<F\;\;$$

The mode shapes and natural frequencies of a pin-pin beam with a proof mass in the middle can be expressed as derived in [65]. In [65], a total of 8 boundary conditions were applied at both ends and mid-span of the beam to obtain the mode shapes and the natural frequencies. Furthermore, Newton’s 2nd law was applied at mid-span to relate the shear forces and bending moments due to the added proof mass. Finally, 8 equations were obtained and expressed in a matrix form. The characteristic equation was obtained by setting the determinant of the matrix to 0. At this stage, the 1st mode shape of a pin-pin beam with a proof mass in the middle can be expressed as in (9). The dimensionless frequency number $\lambda_{1}$ of the 1st mode shape can be obtained by solving the characteristic equation derived in [65].

(9) $$\phi_{1}\left(x\right)=A\;\left[sinh\left(\lambda_{1}x\right)-sin\left(\lambda_{1}x\right)\;\frac{cosh\left(\frac{\lambda_{1}L}{2}\;\right)}{cos\left(\frac{\lambda_{1}L}{2}\right)}\right]$$

The modal amplitude, A in (9) can be solved by using the mode shape orthogonality conditions. Since we are only interested in the 1st mode shape, Equations (10) and (11) are accordingly used to normalize the corresponding mode shape as the following:

(10) $$\int_{0}^{L}\phi_{1}\left(x\right)\;m\;\phi_{1}\left(x\right)dx+\phi_{1}\left(L/2\right)\;M_{t}\;\phi_{1}\left(L/2\right)+\phi_{1}^{\prime }\left(L/2\right)\;I_{t}\;\phi_{1}^{\prime }\left(L/2\right)=1$$

(11) $$\int_{0}^{L}\;\phi_{1}\left(x\right)\;YI\;\phi_{1}\prime \prime \prime \prime \left(x\right)dx-\phi_{1}\left(L/2\right)\;YI\;\phi_{1}\prime \prime \prime \left(L/2\right)+\phi_{1}{\;}^{\prime }\left(L/2\right)\;YI\;\phi_{1}{\;}^{\prime \prime }\left(L/2\right)=\omega^{2}$$

where, the mass and mass moment of inertia of the proof mass are denoted by $M_{t}$ and $I_{t}$, respectively. ω is the 1st undamped natural frequency as indicated in (12).

(12) $$\omega =\lambda_{1}^{2}\sqrt{\frac{YI}{mL^{4}}}$$

The mode shapes and natural frequencies of a simply supported beam without a proof mass obtained from the literature [66] are used to check the validity of the analytical model that is presented in this paper by comparing the resulting mode shapes and natural frequencies when setting the $M_{t}$ and $I_{t}$ to 0 to the ones obtained for the conventional simply supported beam and the results were found to be matching. Furthermore, the approximate formulas for the 1st natural frequency and mode shape for a simply supported beam when a proof mass is added are verified with Blevins [67] as further verification of the analytical model. The governing Equation (5) can be decoupled. This is achieved by pre-multiplying it with the mode shape, ϕ(x), and then integrating over the beam length, L, and then applying the orthogonality conditions as illustrated in (13).

(13) $$\begin{array}{c} \int_{0}^{L}\phi_{j}\left(x\right)\left[\begin{array}{c} m\frac{\partial^{2}}{\partial t^{2}}\sum_{j=1}^{\infty }\phi_{j}\left(x\right)T_{j}\left(t\right)+c\frac{\partial }{\partial t}\sum_{j=1}^{\infty }\phi_{j}\left(x\right)T_{j}\left(t\right)+YI\frac{\partial^{4}}{\partial x^{4}}\sum_{j=1}^{\infty }\phi_{j}\left(x\right)T_{j}\left(t\right)\\ +\left[F_{\mathrm{impact}\;}-\frac{(YA)eq}{2L}\int_{0}^{L}{\left(\frac{\partial }{\partial x}\sum_{j=1}^{\infty }\phi_{j}\left(x\right)T_{j}\left(t\right)\right)}^{2}dx\right]\frac{\partial^{2}}{\partial x^{2}}\sum_{j=1}^{\infty }\phi_{j}\left(x\right)T_{j}\left(t\right)\\ +\vartheta \left[\frac{d\delta (x)}{dx}-\frac{d\delta (x-L)}{dx}\right]V\left(t\right) \end{array}\right]dx=0 \end{array}$$

At this stage, the modal response of the electro–mechanical coupled differential equation is obtained as indicated in (14).

(14) $$\overset{..}{T}\left(t\right)+2\zeta \omega \;\dot{T}\left(t\right)+\left(\omega^{2}+\bar{F}\right)\;T\left(t\right)+G\;T{\left(t\right)}^{3}+\alpha \;V\left(t\right)=0\;$$

In addition, Equation (7) is used in the coupled electrical Equation (6) of the proposed sensing device as indicated in (15) [68].

(15) $$C_{p}\;\dot{V}\left(t\right)+\frac{V\left(t\right)}{R_{l}}=\alpha \;\dot{T}\left(t\right)$$

ζ is the mechanical damping ratio that is proportional to the total equivalent bending stiffness of the composite beam. The terms $\bar{F}$, G, and the coupling coefficient α that are obtained from (13), are calculated as indicated in (16).

(16) $$\begin{array}{c} \bar{F}=F_{impact}\int_{0}^{L}\phi_{1}\prime \prime \left(x\right)\;\phi_{1}\left(x\right)dx\\ G=-\frac{{\left(YA\right)}_{eq}}{2L}\int_{0}^{L}\phi_{1}\prime^{2}\left(x\right)\;\phi_{1}\prime \prime \left(x\right)\;\phi_{1}\left(x\right)dx\\ \alpha =\vartheta \int_{0}^{L}\phi_{1}\left(x\right)\left(\frac{d\delta \left(x\right)}{dx}-\frac{d\delta \left(x-L\right)}{dx}\right)dx \end{array}$$

## 3. The General Characteristics of the Proposed Sensing Device

**Figure 2:**
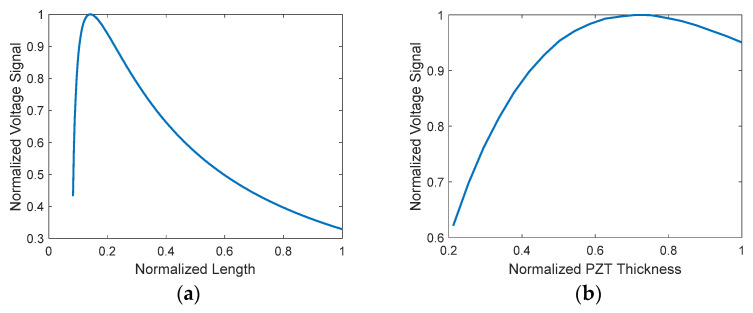
Normalized maximum measured voltage amplitude vs. normalized (**a**) length (**b**) PZT thickness.

This part of the paper investigates the effects of the proposed sensing device’s physical parameters on the measured voltage signal output. The physical parameters included in this study include beam length, L and the thicknesses of the PZT, $h_{p}$. As a rule of thumb, increasing the length of the beam will decrease the total structure stiffness and produce more deflection which results in more voltage output. However, since the proposed device is designed to be excited at its fundamental buckling force, the maximum axial deflection is limited to a certain extent by the value of the rainfall droplet’s impact force. That means, increasing the length beyond a certain point will increase the rigidity of the beam and reduces the effect of the rainfall droplet impact force and consequently lowers the deflection and the measured voltage signal significantly as is seen in Figure 2a. For the same reason, the substructure thickness should be marginally small. In Figure 2a,b, the voltage signal is plotted against a wide range of beam lengths and PZT thicknesses, respectively, by numerically solving Equations (14) and (15) while making sure that the overall stresses do not exceed the maximum yield stress of the composite structure of the proposed sensing device, as indicated in Equation (17). Furthermore, in Figure 2, the rainfall impact force must be larger than the first critical buckling force. Similarly, increasing the thickness of the PZT layers and/or the width of the beam to a certain extent can enhance the voltage signal by increasing the surface area of the active piezoelectric layer just before the piezoelectric patch starts acting rigid, as illustrated in Figure 2b, where the voltage signal is plotted against a wide range of piezoelectric thicknesses. In all possible and un-optimized configurations, the voltage range in Figure 2a,b is [0.2681 to 1.8] and [0.0043 to 0.0259], respectively.

The proposed sensing device is designed to measure the impact forces of the three types of rain. The GA optimization method is employed in the next section to find the optimum configuration of the sensing device in terms of length, width, substrate, and PZT layer thicknesses to maximize the measured voltage signal output. This is accomplished by making sure that the beam is excited at its first buckling load. The physical dimensions are subject to the available fabrication technology rules.

## 4. Optimizing the Proposed Sensing Device Using Genetic Algorithm Technique

**Figure 3:**
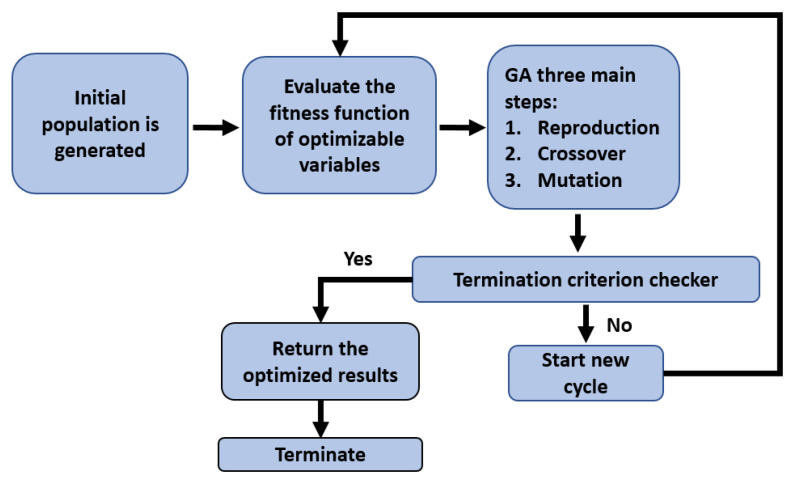
Flowchart of the GA optimization mechanism.

The well-known optimization method based on the genetic algorithm technique (GA) [69], is a well-established technique to obtain very adequate results for real-life optimization problems [70]. The GA can be simply represented in a flowchart that precisely explains the general steps of the mechanism as illustrated in Figure 3.

The first step of the optimization mechanism cycle starts by randomly generating an initial set of the design variables (*n* individuals), called the population. These variables are referred to as chromosomes. In the second step, the ‘fitness function’ is utilized to estimate the corresponding design variables and then they are ranked based on the fitness value they attain. At this point, GA natural selection is utilized where two chromosomes with the highest scores are selected. This brings us to the cross-over stage, where some segments of the selected chromosome strings are exchanged. As a result, new pairs of chromosomes that are identified as offspring are produced. The last step is denoted by the mutation step that occurs at the end of the corresponding optimization cycle. In this step, part of the new offspring binary sequence is swapped from one to zero, or conversely, to guarantee the convergence of the method to a global optimum. This is one of the great advantages of the GA algorithm over other optimization techniques that can be possibly trapped in local optima. At this point, the GA mechanism’s first cycle is completed.

With a newly developed population set, in the next cycle, the GA process including the three main steps is repeated. The process continues until termination criteria are met as indicated in the corresponding flowchart of Figure 3. At that point, the process is concluded, and the optimized values of the design variables are returned along with the estimated objective function. If the termination criteria are not met, the fitness evaluation is conducted again, and the process moves up to a new cycle, and so on. In this work, we aim to maximize the measurable voltage signal V(t) to enhance the measuring capabilities of the proposed device. It must be noted that the sensing device is designed to be excited at its fundamental buckling force. Furthermore, the resulting stresses should not exceed the maximum yield stress of the composite structure of the proposed sensing device. The axial stresses can be expressed as:

(17) $$\begin{array}{c} \sigma_{p}=\frac{{\left(YA\right)}_{p}}{bh_{p}\;\left[2{\left(YA\right)}_{p}+{\left(YA\right)}_{s}\right]}\\ \sigma_{s}=\frac{{\left(YA\right)}_{s}}{bh_{p}\;\left[2{\left(YA\right)}_{p}+{\left(YA\right)}_{s}\right]} \end{array}$$

where $\sigma_{p}$ and $\sigma_{s}$ are the axial stresses experienced by the PZT layer and the substructure of the composite structure of the sensing device, respectively. At this stage, GA optimization can be formulated as:

(18) $$\begin{array}{c} \mathrm{Maximize}:\;\left\{V\right\}\\ \mathrm{Subject}\mathrm{to}\;F_{c}<F_{impact},\;\;\;\;\sigma_{p}<\sigma_{yp}\;,\;\;\;\;\sigma_{s}<\sigma_{yp}\\ \mathrm{Subject to: design rules of the optimizable variables} \end{array}$$

where $\sigma_{yp}$ and $\sigma_{ys}$ are the maximum yield stresses of the PZT and substructure layers, respectively [71]. The design variables are L, b, $h_{p}$, $h_{s}$, and $M_{t}$. A combination of PSI-5H4E piezoelectric and brass materials is used for the composite structure of the proposed sensing device. The values of the design variables are constrained by the available fabrication technology. The load resistance, $R_{l}$ is selected by finding an electric resistance that matches the internal impedance of the composite structure of the proposed device [72]. The optimized design variables, the composite beam material, and electrical properties are listed in Table 2. The optimal load resistance is listed in the 15th row.

## 5. Results and Discussion

**Figure 4:**
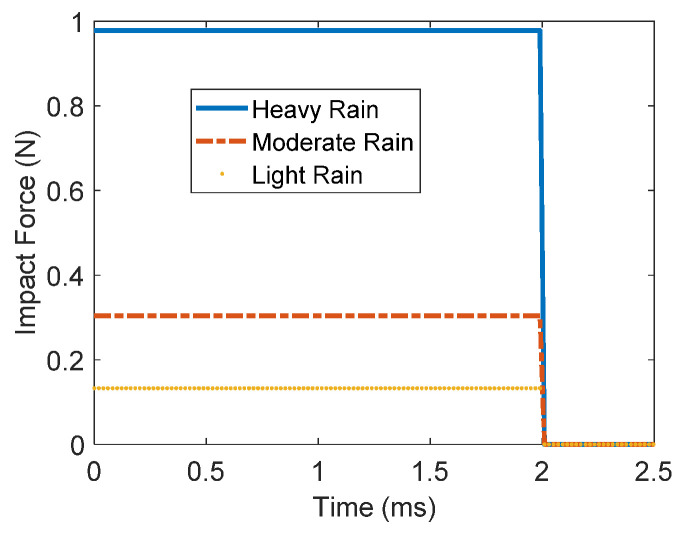
Rainfall droplet’s impact force profile of light, moderate, and heavy rain types.

**Figure 5:**
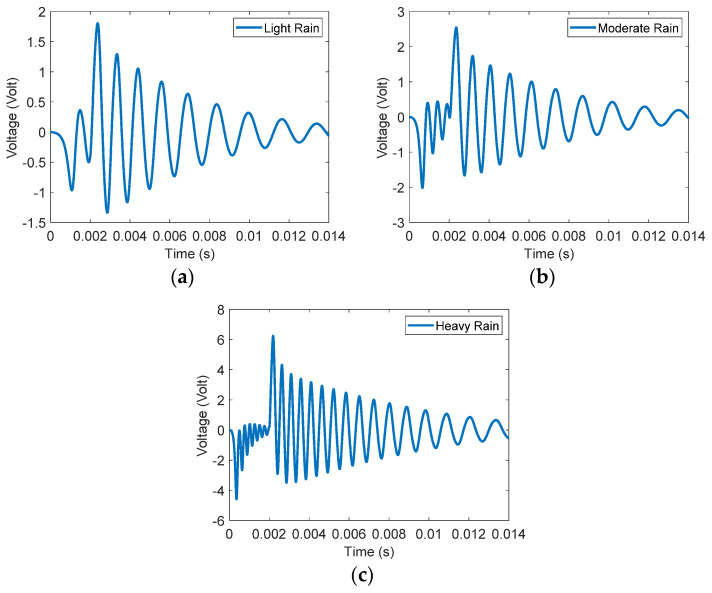
Voltage signal output due to the impact force of (**a**) light rain (**b**) moderate rain (**c**) heavy rain.

To validate the purpose of the proposed rainfall sensing device, the three types of rainfall impact forces listed in Table 1 are investigated. The corresponding rainfall impact force profiles are illustrated in Figure 4. As it is discussed in this paper, the time duration of the droplet’s impact force to rise and disappear is almost 2 ms and the forcing profile takes the shape of a step function. At this stage, Equations (14) and (15) are employed to generate the time-domain response of the measured voltage signal of the proposed self-sensing device. The nonlinear Equation (14) can be solved analytically with several methods, including the extended Galerkin method, where the nonlinear equation of flexure of an elastic beam can be solved for a highly accurate solution and accurate prediction on nonlinear structural behavior [73,74]. In this paper, we use MATLAB to numerically solve Equations (14) and (15) and to generate the time-domain response of the output voltage signals of the three types of rainfall, as indicated in Figure 5. The numerical analysis by MATLAB showed very accurate and efficient results. The plots in Figure 5 show two states of the beam: state one, when the beam is under the influence of the impact force of a period of 2 ms that corresponds with an impact frequency of 500 Hz; and state two, when the impact force disappears and the beam starts vibrating at its first natural frequency.

The voltage mean square root, $V_{rms}$ of the sensing device for the three types of rainfall are listed in the second column of Table 3. The third and fourth columns indicate the rainfall impact force and the ratio of the impact force of the first buckling load of the proposed sensing device.

**Figure 6:**
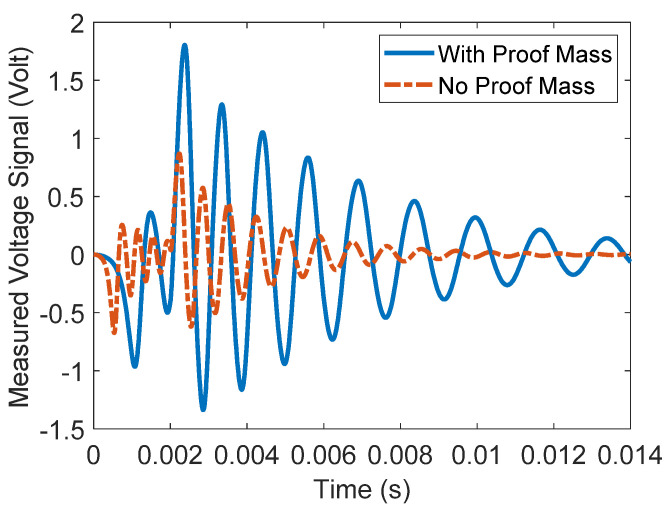
Voltage signal output by the proposed sensing device with and without proof mass.

To further investigate the proposed rainfall sensing device, the results are briefly compared with those of a sensing device that consists of a beam without a proof mass in the middle. The comparison is based on the magnitude of measurable voltage signals by the impact forces of the three types of rain. The comparison is listed in Table 4. As can be seen, the measured voltage signals to some extent are enhanced when a proof mass is added to the middle of the beam. In addition, the measured voltage signals by the sensing device with and without added proof mass due to the impact force of the light rain are side by side compared with each other as illustrated in Figure 6. As can be seen, the overall measured voltage signal is enhanced when a proof mass is added to the middle of the beam. Furthermore, it clearly can be seen that the time of oscillation after the impact force disappears (state two) is longer than the time of oscillation with no mass. This is because adding a mass has reduced the first natural frequency of the beam and hence increased the time of oscillation.

**Figure 7:**
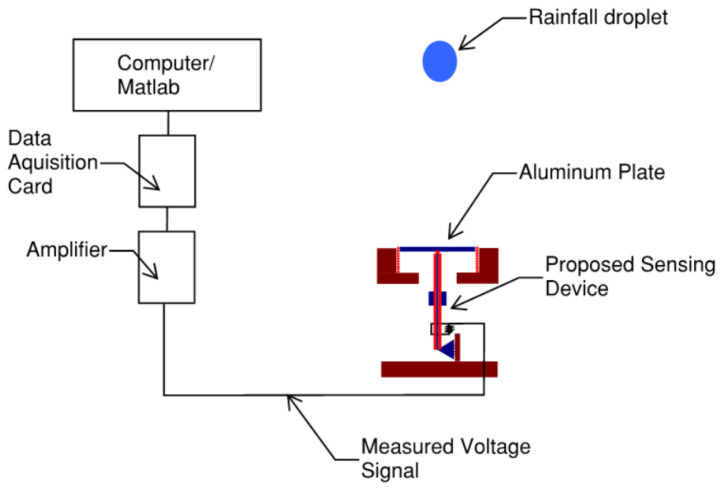
Schematics diagram for a suggested future experiment setup.

A future experiment is highly suggested to validate the analytical model. The measured voltage signal is due to the collision of the rainfall droplet with the aluminum plate. The plate area must be big enough to make sure that the whole collision process occurs on the plate and must be light enough to not affect the recorded measurements. The measured voltage signal can be amplified using an amplifier. The amplified voltage signal can be transferred to a personal computer via a high-accuracy data acquisition board for further analysis. The measured and amplified voltage can be analyzed by MATLAB using the corresponding analytical model to obtain the impact forces by numerically solving the differential Equations (14) and (15). Figure 7 shows a mock-up schematic diagram for the proposed future experimental setup.

**Figure 8:**
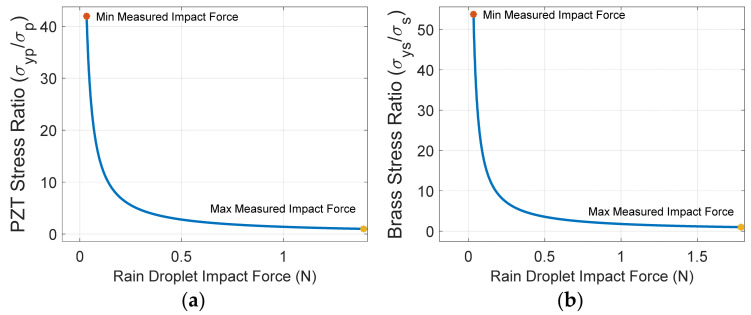
Upper and lower bounds of the measured impact forces of the proposed sensing device.

It is very important to determine the range of the impact forces the rainfall sensing device can measure. The proposed sensing device’s maximum measuring resolution is determined by the maximum impact force it can measure before it breaks due to the excessive axial stress on the substructure and/or piezoelectric layers. The normal stress in the substructure and PZT layers can be calculated from (17). The ratio of the maximum yield stress to the normal stress of the substructure and PZT layers are denoted by $\frac{\sigma_{ys}}{\sigma_{s}}$ and $\frac{\sigma_{yp}}{\sigma_{p}}$, respectively. This ratio must be larger than unity so that the proposed sensing device does not break under the influence of excessive rainfall impact force. Furthermore, the minimum measuring resolution of the proposed sensing device is determined when the measured impact force equals the first buckling load of the device. Figure 8 shows the minimum and maximum impact forces that can be measured by the proposed sensing device. The sensing device resolution is determined by the maximum allowed yield stress ratio of the PZT layer and the substructure as indicated in Figure 8a,b, respectively. It can be seen that the minimum impact force that can be measured is the same for both substructure and PZT layers and equals 0.0332 N. The maximum measured impact force is dominated and determined by the allowable yield stress ratio of the PZT layers and equals 1.451 N. This is because PZT layers have less structural strength when compared to brass. In summary, the sensing device impact force measuring sensitivity is between 0.0332 to 1.395 N and the proposed sensing device is subject to mechanical failure if the impact force is larger than the upper bound of the measuring sensitivity. Furthermore, no voltage signal can be measured if the impact force is less than the lower bound of the device impact force measuring sensitivity.

**Figure 9:**
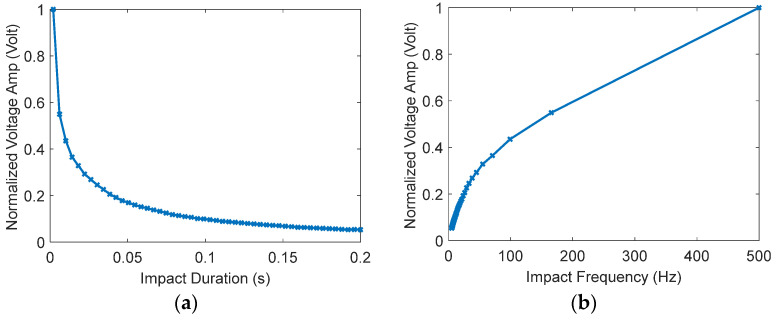
Variation of the voltage signal amplitude with the rain droplet’s impact on (**a**) duration and (**b**) frequency.

At this point, it is necessary to illustrate the general effect of the amount of the impact force and its frequency (impact duration) on the voltage signal. A large amount of impact force will increase the amplitude of the free oscillations of the beam and thus increases the amplitude of the voltage signal, as can be seen in Figure 5. Furthermore, if the duration of the impact force increases (low frequency), the vibration amplitude decreases, and consequently the peak of the voltage signal decreases. The variation of the voltage signal amplitude with the impact duration can be seen in Figure 9a, which ranges from 2 ms to 0.2 s. In Figure 9b, the impact frequency that corresponds with the impact duration is illustrated and ranges from 5 Hz to 500 Hz. Therefore, the best voltage signal is achieved when there is a high frequency (low impact duration) and a high amount of impact force. Our proposed sensing device is optimized for an impact duration of 2 ms. Therefore, it must be noted that at a different impact duration, the GA optimization discussed previously must be repeated to achieve the optimum performance, the optimal design variables, and the resulting natural frequency of the proposed sensing device.

To conclude this section, the energy transfer efficiency  η of the proposed sensing device is estimated. The efficiency can be defined as the ratio of the net output electrical energy $E_{out}$, by the net input mechanical energy denoted by the mechanical work, W. Methods for solving for $E_{out}$ and W in the energy harvesting literature are very sparse. They are highly dependable on the type of problem being studied. $E_{out}$, W, and η are expressed here with the help of reference [75]. Therefore, $E_{out}$ and W can be defined as

(19) $$\;\;\;\;\;\;\;\;\;\;\;\;\;\;\;\;\;\;\;\;\;\;\;\;\;\;\;\;\;\;\;\;\;\;\;\;\;\;\;\;\;\;\;\;\;\;\;\;\;\;\;E_{out}=\int_{0}^{\tau }P_{out}\;dt=\tau \;\frac{V_{rms}^{2}}{\;R_{l}},\;\;\;\;\;\;\;\;\;\;W=\int_{0}^{\tau }P_{in}\;dt=\tau \;m\;\int_{0}^{\tau }A\overset{..}{T}\left(t\right)\;A\dot{T}\left(t\right)\;dt/\tau \;$$

where $P_{out}$ is the power consumed by the external resistor. $P_{in}$ is the net input mechanical power. τ is the signal cycle period as indicated in Figure 5. The voltage mean square root, $V_{rms}$ is listed in Table 3. The input mechanical energy is proportional to the mass and the response of the acceleration and velocity. The functions $\dot{T}\left(t\right)$ and $\overset{..}{T}\left(t\right)$ can be found by solving Equations (14) and (15). The modal amplitude  A is found in (9). As discussed above, the efficiency η can be now estimated as the ratio of $E_{out}$ by W as illustrated in Equation (20).

(20) $$\eta =\frac{\;E_{out}}{W}$$

At this point, η of the proposed sensing device for the three types of rainfall can be calculated as listed in Table 5.

The results presented in Table 5 agree with our findings in Table 3. The droplet’s impact force in the moderate and heavy rainfall types surpasses not only the first critical load but also higher critical loads, and thus higher buckling modes are excited. In the light rainfall type, only the first buckling mode is excited. Due to the symmetry of the higher buckling modes, there are high electric charge cancelations associated with higher modes [76].

## 6. Verification Study of the FE COMSOL Model

In this section, a finite element model (FEM) is constructed to verify the analytical results. An FE model is developed using a commercial FEM software known as COMSOL 6.0 Multiphysics that is used to simulate the results numerically. The COMSOL is vastly used in finite element analysis due to its reliability and accuracy to simulate complicated real-life environmental problems that are difficult to experimentally or analytically establish. Several studies have been conducted in previous research to numerically verify the experimental results using COMSOL, which shows the reliability of our FEM results.

We begin our finite element analysis by finding the optimum mesh distribution to enhance the convergence factor when simulating the model to find the first natural frequency of the proposed sensing device. Finding the optimum mesh convergence provides the confidence, consistency, and validation of the finite element (FE) COMSOL model. As a result, Table 6 compares the finite element method (FEM) and the analytical model based on the first natural frequency of both scenarios with and without a proof mass. The table reveals an excellent convergence between the two methods with a maximum error=0.93%.

**Figure 10:**
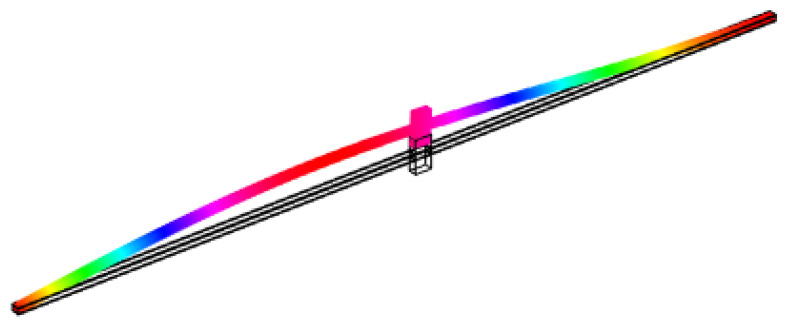
First buckling mode of the proposed sensing device.

Furthermore, FEM is used to simulate the first buckling mode shape as illustrated in Figure 10. Consequently, Table 7 is constructed to compare the ratio of light rain impact force on the first buckling load of the proposed sensing device obtained analytically and numerically, respectively. The critical load factor indicates the number by which the load must be multiplied until the model under the associated load becomes unstable (buckling).

**Figure 11:**
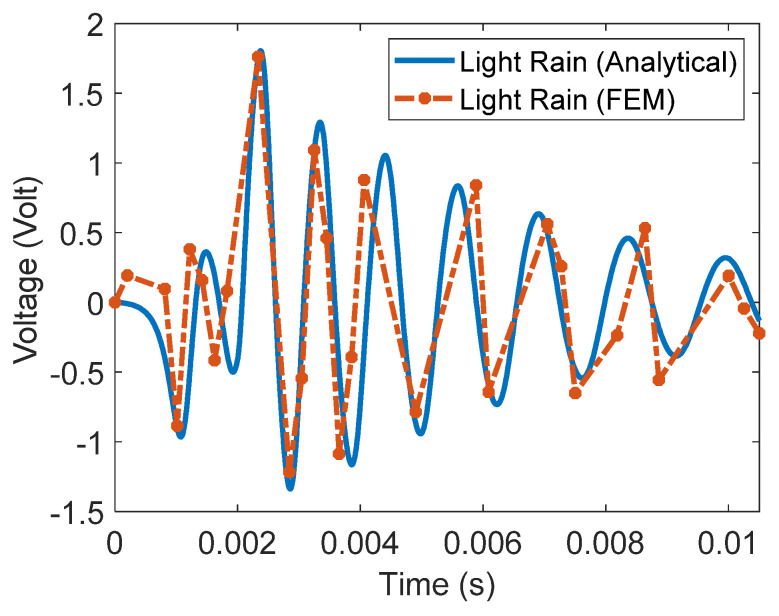
Analytically and numerically measured voltage signal by the proposed rainfall sensing device.

To further verify the analytical model, the measured voltage signal obtained analytically is verified numerically using COMSOL 6.0 software. Figure 11 illustrates the time-domain response of the measured voltage signal due to the impact force of the light rainfall droplet for both models, the analytical and the FE models, respectively. It can be concluded from the figure that both models are in good agreement with each other. Nevertheless, the deviation between the analytical and FEM results can be justified, as part of the high nonlinear behavior of the analytical model could not be captured and properly simulated by the proposed FE model. Furthermore, when very small physical dimensions are involved, as is the case here, FEM model convergence can be affected significantly.

## 7. Conclusions

In this paper, a proposed sensing device was utilized to measure the peak impact forces of rainfall droplets via the direct effect of piezoelectric material. Piezoelectric sensors and transducers proved to be the best option for experimental measurements due to their high-frequency response and high sensitivity. The voltage signal is generated due to the impact force of the rainfall droplet collision on very light and thin aluminum. The proposed sensing device was composed of a bimorph composite-piezoelectric beam that buckles due to the effect of the rain droplet’s vertical impact force. The proposed device was designed for optimal performance in terms of the amount of voltage that can be measured. The genetic algorithm (GA) automated optimization method was used to find the optimal parameters of the proposed sensing device that were constrained by: (1) the resulting stresses did not exceed the maximum yield stress of the composite structure of the proposed sensing device, and (2) the sensing device was excited at its fundamental buckling force. A proof mass was added to the middle of the beam to amplify the magnitude of the measured voltage signal. There was a noticeable increase in the RMS values of the output voltage signals compared to when no proof mass was added. Furthermore, the general characteristics of the proposed sensing device’s physical parameters (including the length and the thickness of the beam) and its effect on the measured voltage signal output were investigated. The sensing device impact force measuring sensitivity (resolution) was determined to be between 0.0332 to 1.395 N. The resolution was determined by the minimum impact force needed to excite the beam at its first buckling mode and by the largest impact force before the proposed sensing device became subject to mechanical failure. The energy transfer efficiency  η of the proposed sensing device for the three types of rainfall was estimated. The output voltage signal was recorded by the computer via the data acquisition system. The output voltage signal could be transferred to a personal PC for further analysis and processing. The analytical model of the proposed device was derived using Euler–Bernoulli thin beam theory. The analytical model was then verified numerically using the FE model developed by the COMSOL 6.0 Multiphysics commercial software and the analytical and numerical results were found to be in good agreement.

## Figures and Tables

**Table 1:** Rainfall droplet physical information.

Rain Type	Size (mm) [44]	Terminal Velocity (m/s) [45,46]	Impact Force (N) [48,49]
Light	2.0	6.49	0.133
Moderate	2.6	7.57	0.3042
Heavy	4.0	8.83	0.978

**Table 2:** Optimized design variables and the proposed rainfall sensor physical properties.

Parameter	Description	Size
L	Beam length	10 mm
b	Beam width	0.10 mm
$h_{p}$	PZT film thickness	0.0143 mm
$h_{s}$	Substrate thickness	0.053 mm
$\rho_{s}$	Substrate density	9000 kg/m^3^
$\rho_{p}$	Piezoelectric density	7800 kg/m^3^
$Y_{s}$	Substrate modulus of elasticity	105 Gpa
$Y_{p}$	PZT modulus of elasticity	61 Gpa
$\sigma_{ys}$	Substrate max yield stress	255 Mpa
$\sigma_{yp}$	PZT max yield stress	114.8 Mpa
$d_{31}$	Piezoelectric constant	−274 pm/v
$\varepsilon_{33}^{s}$	PZT permittivity constant	25.5 nF/m
$M_{t}/\mathrm{mL}$	Proof mass-to-beam mass ratio	1.51
$R_{l}\;$	Electric resistive load	8.2 kΩ

**Table 3:** Proposed rainfall sensing device response to the three types of rainfall.

Rain Type	$V_{rms}$ (V)	Impact Force (*N*)	$\frac{F_{impact}}{\;F_{c}}$	# of Modes Excited
Light	0.64	0.133	3.99	1
Moderate	0.862	0.3042	9.16	3
Heavy	1.8	0.978	29.45	5

**Table 4:** Output voltage magnitude of the sensing device with and without proof mass.

	With Proof Mass	Without Proof Mass
Rain Type	$V_{rms}\;\left(V\right)$	$V_{rms\;}\left(V\right)$
Light	0.64	0.26
Moderate	0.862	0.6
Heavy	1.8	1.16

**Table 5:** Energy transfer efficiency of the sensing device for the three types of rainfall.

Rain Type	Energy Transfer Efficiency, η
Light	2.6%
Moderate	1.9%
Heavy	0.8%

**Table 6:** Natural frequency comparison between FEM and analytical models.

	First Natural Frequency (Hz)	
	No Mass	With Mass
Analytical	1089	540.95
FEM	1078.8	536.15
Error [] %	0.93	0.8

**Table 7:** First critical buckling load ratio comparison between FEM and analytical models.

	$\mathrm{Buckling}\;\mathrm{Load}\;\mathrm{Ratio}\;\frac{F_{impact\left(light\;rain\right)\;\;}}{\;F_{c}}$
Analytical	0.24
FEM	0.232
Error [] %	3.3

## Data Availability

Not applicable.
